# A dose-response based model for statistical analysis of chemical genetic interactions in CRISPRi libraries

**DOI:** 10.1101/2023.08.03.551759

**Published:** 2023-08-05

**Authors:** Sanjeevani Choudhery, Michael A. DeJesus, Aarthi Srinivasan, Jeremy Rock, Dirk Schnappinger, Thomas R. Ioerger

**Affiliations:** 1 Department of Computer Science and Engineering, Texas A&M University, College Station, Texas, United States of America; 2 Laboratory of Host-Pathogen Biology, The Rockefeller University, New York, New York, United States of America; 3 Department of Microbiology and Immunology, Weill Cornell Medical College, New York, New York, United States of America

## Abstract

An important application of CRISPR interference (CRISPRi) technology is for identifying chemical-genetic interactions (CGIs). Discovery of genes that interact with exposure to antibiotics can yield insights to drug targets and mechanisms of action or resistance. The premise is to look for CRISPRi mutants whose relative abundance is suppressed (or enriched) in the presence of a drug when the target protein is depleted, reflecting synergistic behavior. One thing that is unique about CRISPRi experiments is that sgRNAs for a given target can induce a wide range of protein depletion. The effect of sgRNA strength can be partially predicted based on sequence features or empirically quantified by a passaging experiment. sgRNA strength interacts in a non-linear way with drug sensitivity, producing an effect where the concentration-dependence is maximized for sgRNAs of intermediate strength (and less so for sgRNAs that induce too much or too little target depletion). sgRNA strength has not been explicitly accounted for in previous analytical methods for CRISPRi. We propose a novel method for statistical analysis of CRISPRi CGI data called CRISPRi-DR (for Dose-Response model). CRISPRi-DR incorporates data points from measurements of abundance at multiple inhibitor concentrations using a classic dose-response equation. Importantly, the effect of sgRNA strength can be incorporated into this model in a way that mimics the non-linear interaction between the two covariates on mutant abundance. We use CRISPRi-DR to re-analyze data from a recent CGI experiment in *Mycobacterium tuberculosis* and show that genes known to interact with various anti-tubercular drugs are ranked highly. We observe similar results in MAGeCK, a related analytical method, for datasets of low variance. However, for noisier datasets, MAGeCK is more susceptible to false positives whereas CRISPRi-DR maintains higher precision, which we observed in both empirical and simulated data, due to CRISPRi-DR’s integration of data over multiple concentrations and sgRNA strengths.

## Author Summary

CRISPRi technology is revolutionizing research in various areas of the life sciences, including microbiology, affording the ability to partially deplete the expression of target proteins in a specific and controlled way. Among the applications of CRISPRi, it can be used to construct large (even genome-wide) libraries of knock-down mutants for profiling antibacterial inhibitors and identifying chemical-genetic interactions (CGIs), which can yield insights on drug targets and mechanisms of action and resistance. The data generated by these experiments (i.e., nucleotide barcode counts from high throughput sequencing) is voluminous and subject to various sources of noise. The goal of statistical analysis of such data is to identify significant CGIs, which are genes whose depletion sensitizes cells to an inhibitor. In this paper, we show how to incorporate both sgRNA strength and drug concentration simultaneously in a model (CRISPRi-DR) based on an extension of the classic dose-response (Hill) equation in enzymology. This model has advantages over other analytical methods for CRISPRi, which we show using empirical and simulated data.

## Introduction

CRISPR interference (CRISPRi) has become popular for genome-wide profiling of the biological roles of genes in various growth conditions. By detecting growth defects caused by depletion of individual genes or operons, genes may be associated with responses to different stress conditions. The concept of gene ‘vulnerability’ has recently been introduced to describe the sensitivity of cells to partial depletion of individual proteins. By this definition, highly vulnerable genes are genes for which minimal depletion of protein levels causes growth impairment, which can be quantified efficiently on a genome-wide scale using high-throughput sequencing [1]. The vulnerability of a gene can be condition dependent, or strain dependent [1]. CRISPRi can be used to reveal targets of antibiotics or mechanisms of resistance through chemical-genetic interactions [2, 3]. CRISPRi libraries are often designed to contain multiple small guide RNAs (sgRNAs) targeting each gene, resulting in a population of thousands of individual depletion mutants [1]. The abundance of each sgRNA can be quantified by amplifying the sgRNA targeting sequence which functions as a molecular barcode, and then performing deep sequencing to count the number of barcodes for each sgRNA in a treatment. The analysis of such datasets is challenging, due to various sources of noise, which introduces variability in the counts.

A previously published method for analyzing CRISPRi datasets, called MAGeCK [4], fits the data to a negative binomial distribution, calculates a log-fold-change (of mean counts) for each gene between a treatment condition and a reference condition (control, e.g. buffer with 5% DMSO as solvent), and uses a negative binomial (NB) mass function to test the differences in significance of sgRNA abundance between treatments and controls. To evaluate effects at the gene level, individual sgRNAs are combined in MAGeCK using Robust Rank Aggregation (RRA) to prioritize genes whose sgRNAs show greater enrichment or depletion on average than other genes in the genome. MAGeCK has been used for evaluating chemical-genetic interactions (CGI) with antibiotics [4].

However, MAGeCK has two limitations for this application. First, gene-drug interaction studies are usually carried out over several drug concentrations around the MIC (minimum-inhibitory concentration), since it is often difficult to anticipate what concentration will stimulate 50% growth inhibition of mutants in combination with CRISPRi-induced depletion of target proteins. However, MAGeCK analyzes the data for each drug concentration independently (each concentration compared to a no-drug control). Knock-down mutants might exhibit depletion at one concentration but not others. Results from multiple concentrations must be combined post-hoc, such as by taking the union of MAGeCK hits at any concentration. Due to the noise in these CRISPRi experiments, this increases the risk of detecting false positives (in the sense that non-interacting genes that might be mistakenly called as hits independently at different concentrations are combined). In practice, for some datasets, MAGeCK reports an unreasonably large set of significant interactions, not all of which may be biologically genuine. Second, MAGeCK does not explicitly take into account differences in sgRNA strength. Different sgRNAs are known to induce different degrees of depletion of their target genes. This can be quantified beforehand by evaluating the growth rate of individual mutants in a passaging experiment and determining how fitness correlates with target knockdown [1]. In highly vulnerable genes, the strength or effectiveness of depletion by sgRNAs can span a range from no effect to severe growth defect. This information was not anticipated at the time MAGeCK was developed (as the early applications of CRISPRi were primarily being used to fully inactivate genes, rather than to produce graded effects), and the Robust Rank Aggregation method treats all sgRNAs in a gene as "equal", without differentiating them based on the expected effects due to sgRNA strength.

In this paper, we propose a new methodology for statistical analysis of CRISPRi libraries and identification of chemical-genetic interactions. A regression model is used to integrate data over multiple drug concentrations. The degree of a gene-drug interaction is reflected by the coefficient (or slope) for the dependence of sgRNA abundance on drug concentration. This regression approach was previously introduced for analysis of hypomorph libraries (where there is just one to three mutants representing each gene) [5]. It was based on the theory that depletion of the target of a drug should synergize with increasing concentrations of the drug. While exposure to sub-MIC levels of an inhibitory compound will challenge the growth of all the mutants in a population (hypomorph library), mutants with depletion of a gene that interacts with a drug (e.g. prototypically, an essential gene that is the drug target) will exhibit excess depletion relative to others in the population due to the combined effect of both the growth-inhibition due to the drug treatment in conjunction with the growth-impairment due to knock-down of an essential gene, making these mutants even more sensitive to the drug. For genes that genuinely interact with a given drug, this depletion effect should be exacerbated at higher drug concentrations (i.e. be dose-dependent); genes of greatest relevance are those that exhibit concentration-dependent effects. While the (log of) abundance of an sgRNA does not have to decrease perfectly linearly with the (log of) concentration to obtain a significant negative coefficient (slope) in the regression, there should be a general trend supporting that abundance decreases as concentration increases. Other researchers have exploited CRISPRi in different ways to detect this synergistic behavior for identifying chemical-genetic interactions. For example, the expression of an active form of dCAS9 was titrated to produce different levels of expression of essential proteins in *S. pyrogenes*, looking for genes whose depletion shifted the MIC to inhibitors [3].

One of the challenges in extending this prior regression approach to CRISRPi libraries was incorporating information on sgRNA strengths. Even in essential genes, some sgRNAs may produce strong depletion of the target, while others might be almost completely ineffective, generally depending on sequence attributes (similarity to optimal PAM sequence (protospacer-adjacent motif), length, GC content, etc.) [6]. While sgRNA strength can be partially predicted (with intermediate accuracy) from sequence alone, sgRNA strength can also be empirically quantified by measuring or extrapolating log2-fold-changes of abundance (LFCs) in standard growth media *with* versus *without* induction of CRISPRi at a fixed number of generations [1]. Although one could contemplate adding the strength of each sgRNA (predicted, or empirically measured) into the regression model to predict abundances for each gene, a significant problem (expanded upon below) is that sgRNAs of different strength can show different concentration dependence.

In this paper, we propose a modified regression approach for CRISRPi data (called CRISPRi-DR) that incorporates both drug concentration and sgRNA strength. The approach is based on the classic dose-response (DR) model for inhibition activity of drugs; the activity of a target protein typically transitions from high to low in shape of an S-curve as concentration increases (on a log scale), which can be modeled with a Hill equation. The parameters of the Hill equation for a given drug can be fit by performing a log-sigmoid transformation of the enzyme activity data and then using ordinary least-squares regression. We show how sgRNA strength can be incorporated into this model as a multiplicative effect in the Hill equation, which becomes an additive effect in the log-sigmoid transformed data. The important consequence of this model is that it decouples the concentration-dependence from the sgRNA strength, so they can be fit as independent (non-interacting) terms in the regression. We demonstrate the value of the CRISPRi-DR analysis method by re-analyzing the data from a recent paper using CRISPRi for chemical-genetic interactions to identify targets of antibiotics in *M. tuberculosis*.

## Methods

CRISPRi experiments involve using high-throughput sequencing to tabulate counts of nucleotide barcodes representing abundance of individual mutants in a population (or library). Each mutant has an sgRNA mapping to a target gene that can reduce its expression (when induced with ATC, anhydrotetracycline). In CGI applications, the library is sequenced in the presence of antibiotics or inhibitors at various concentrations, along with a no-drug control. If $Y_{ijk}$ is the abundance (i.e. count) for an sgRNA i in a condition j for replicate k, normalized abundance can be given by $Y_{ijk}^{\prime }=\frac{Y_{ijk}}{\sum_{x=1}^{n}Y_{xjk}}$, where each count is divided by the sum of counts of the n sgRNAs observed in a given condition and replicate. Let $U_{\;i}^{\prime }$ be the normalized abundance of sgRNA i in the uninduced (−ATC) library, then the normalized relative abundances of an sgRNA i in all induced (+ATC) samples can be calculated as: $A_{ijk}=\frac{Y_{ijk}^{\prime }}{U\prime_{i}}$, assuming that the abundance in −ATC represents no depletion (100% full abundance). Although increases greater than 1 are possible in treated conditions, these relative abundances ideally range between 0 and 1 (i.e., 100% as a percentage). This absolute scale is required for the dose-response model.

### CRISPRi dose-response model

The CRISPRi-DR model for analyzing CRISPRi data from CGI experiments is an extension of the basic dose-response model, extended to incorporate sgRNA strengths. The dose-response effect of an inhibitor on the activity of an enzyme is traditionally modeled with the Hill-Langmuir equation.

[1] $$\theta =\frac{1}{1+{\left(\frac{K_{A}}{\left[L\right]}\right)}^{n}}$$

where θ is the fraction of abundance (relative to no drug), [L] is the ligand concentration, $K_{A}$ is the concentration at which there is 50% activity and n is the Hill coefficient.

Applying [1] to the CGI data, the relative abundance of sgRNAs $A_{ijk}$ is used as the predictor variable and $\left[D_{j}\right]$ is the concentration of drug j that the kth replicate count of sgRNA i was extracted from,

[2] $$A_{ijk}=\frac{1}{1+{\left(\frac{EC_{50}(D_{j})}{\left[D_{j}\right]}\right)}^{H_{d}}}$$

The unknown parameters are the ${\text{EC}}_{50}$ value (effective concentration that causes 50% growth inhibition) and the Hill coefficient $H_{d}$. The plot of the concentration versus relative abundance of an sgRNA ($A_{ijk}$) produces a sigmoidal curve, demonstrating how activity decreases as concentration increases, with the ${\text{EC}}_{50}$, representing the mid-point of the transition.

The dose-response model seen in [2] can be extended to account for sgRNA strength by incorporating a multiplicative factor in the denominator:

[3] $$A_{ijk}=\frac{1}{1+{\left(\frac{EC_{50}(D_{j})}{\left[D_{j}\right]}\right)}^{H_{d}}{\left(\frac{K_{s}}{S_{i}}\right)}^{H_{s}}}$$

sgRNA strength, $S_{i}$, is quantified by the estimate degree of growth impairment at 25 generations of growth in-vitro (log2-fold-change of abundance with ATC vs without, $LFC=log2(\frac{+ATC}{-ATC})$ in the absence of drug, extrapolated from a model fit to empirical data from passaging for each sgRNA [1]. $K_{s}$ represents the unknown intermediate sgRNA strength that causes 50% depletion of mutant abundance (half-way between no depletion and full depletion), and the $H_{s}$ is the unknown Hill coefficient that represents how sensitive mutant abundance is to depletion of the target protein.

### Relationship between drug concentration and gene depletion within the CRISPRi-DR model

**Fig 1.**
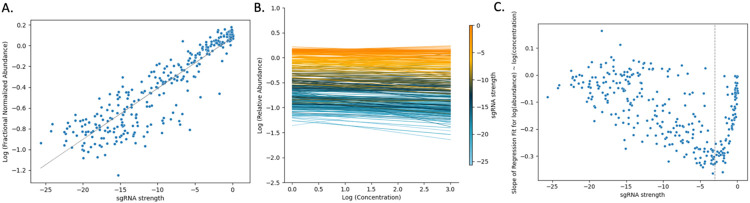
Effect of sgRNA strength and drug concentration on abundance of mutants in *rpoB* in a CRISPRi library treated with RIF (D5). (A) Comparison of fractional abundances of sgRNAs in *rpoB* (+ATC / −ATC) to their strengths (in the form of extrapolated LFCs 25 generations in the future). There is a strong correlation of depletion and sgRNA strength in rpoB (RNA polymerase beta subunit, target of rifampicin). There is a linear relationship between these two values, evident by the line of best fit (R^2^ = 0.82). Since strength is measured as extrapolated LFC, the more negative the LFC, the stronger the sgRNA. Here we see that almost linearly, as sgRNA strength increases, abundance decreases. (B) Regression lines for log(relative abundance) against log(concentration) for all sgRNAs in *rpoB* in a library treated with RIF D5. Although the starting abundance varies, the majority of the regression lines show a negative slope, demonstrating that as concentration of RIF increases, the abundance of sgRNAs in *rpoB* decrease. The lines that reflect the extremes of the sgRNA strength (orange or blue), are flat and do not show much change in abundance. Comparatively, the middle of sgRNA strength range (navy blue) show the greatest negative slopes reflecting this is the region of ideal sgRNA strength. (C) Comparison of sgRNA strength and slopes of a regression of log(relative abundance) against log(concentration) for each sgRNA in *rpoB* in a library treated with RIF D5. Each slope (one for each sgRNA) seen in Panel B versus its strength show a parabolic curve. The strongest sgRNAs (left on the plot) and the weakest sgRNAs (right side on the plot) show slopes around 0. These regressions are the flat lines in at the top and the bottom of the graph in Panel B. As seen in Panel A, with strong sgRNAs (left of plot), we already have a low starting abundance, so with increasing concentration, there is little depletion. With weak sgRNAs (right of the plot), starting abundances are high, but the sgRNA is too weak to show depletion with increasing concentration. The minimum of the parabolic curve (dotted line) are sgRNAs of intermediate strength where the ability to detect synergy with the drug is maximized

Abundance of mutants in a CRISPRi CGI experiment can be affected simultaneously by both presence of an inhibitor and depletion of a vulnerable gene. However, the concentration-dependent effect of a drug on mutant abundance can be different for sgRNAs of different strength. For example, a strong sgRNA can cause excessive depletion, making it difficult to detect additional decreases due to increasing drug concentration; weak sgRNAs might not induce enough depletion to synergize with the drug; sgRNAs of intermediate strength can provide just the right amount of depletion to maximize the interaction with the drug, producing the most pronounced concentration-dependent effects (sensitization). Fig 1 illustrates this with sgRNAs, spanning a range of strengths, in *rpoB* (RNA polymerase beta subunit, target of rifampicin) treated with rifampicin (RIF) over a range of concentrations. In Fig 1A , the sgRNA strength (extrapolated LFCs at 25 generations) is plotted versus observed depletion (log of +ATC/−ATC) in the absence of any drug for each sgRNA in *rpoB* in a log-log space. Since strength is measured as extrapolated LFC, the more negative the LFC, the greater the depletion and hence stronger the sgRNA. The points follow the linear dashed line, demonstrating that, as sgRNA strength increases, abundance decreases. The lines in Fig 1B are regression fits obtained for each sgRNA in *rpoB* in RIF (5 days of pre-depletion, D5) using regression of log abundances with log concentration, $\log(A_{ijk})=C+B\cdot \log([D_{j}])$ , where C is in the intercept and B is the slope of the regression, representing concentration dependence, and $\log(A_{ijk})$ are log relative abundances obtained as described above. The left-most side of Fig 1B (log concentration = 0) shows the range of abundances with no drug concentration (ATC-induced library in buffer). Regression lines have starting points at various abundances (relative to −ATC), due solely to the growth impairment cause by depleting *rpoB*. As concentration of RIF increases, some of the sgRNAs show very negative slopes, while other sgRNAs show slopes closer to 0. This illustrates that sgRNAs within a gene in a particular condition can show vastly different concentration dependencies. A parabolic-type curve emerges in Fig 1C when the slopes from the regressions performed on each sgRNA seen in Fig 1B are plotted against the sgRNA strengths. The strongest sgRNAs (left on the plot) and the weakest sgRNAs (right side on the plot) show slopes around 0. These regressions represent the flat lines in at the top and the bottom of the graph in Fig 1B . As seen in Fig 1A, strong sgRNAs (left of plots Fig 1A and Fig 1C) already have a low starting abundance, so with increasing concentration, there is little depletion. With weak sgRNAs (right of plots in Fig 1A and Fig 1C), starting abundances are high, but the sgRNAs are too weak to show depletion with increasing concentration. The sgRNAs surrounding the minimum point of this parabolic curve (dashed line) reflect those of intermediate strength, where the ability to detect synergy with the drug is maximized. Similar behavior is observed for many other genes in the presence of other drug treatments. The strength where the slopes reach their extrema points can be different for each gene. The variability of concentration-dependence (slope) with sgRNA strength suggests a possible non-linear interaction between the variables. However, this nonlinearity is captured in the multiplicative terms of the dose-response model (Eqn. 3).

## Linearization and parameter estimation

The dose-response model [3] can be linearized through a log-sigmoid transformation.


[4] $$\log\left(\frac{A_{ijk}}{1-A_{ijk}}\right)=H_{d}\cdot \log([D_{j}])+H_{s}\cdot S_{i}+C\;\;C=H_{s}\cdot \log(K_{s})-H_{d}\cdot \log\left(EC_{50}(D_{j})\right)$$

**Fig 2.**
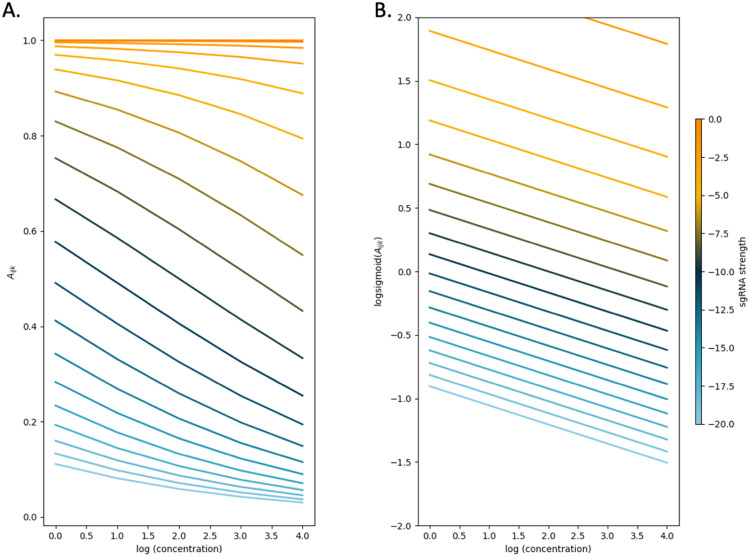
The log-sigmoid transformation of abundances allows the CRISPRi-DR model to factor in the non-linear effect of sgRNA strength on concentration dependence. (A) Simulation of sgRNAs abundances for an ideal essential gene. Parameters used in simulation: $H_{s}=-4$, ${\text{EC}}_{50}=0.25$, $K_{s}=-10$ and $H_{d}=-0.5$ over a range of sgRNA strengths and drug concentrations. (B) When the log-sigmoid transformation of the abundances is applied, we see all the regression fits are parallel to one another, allowing to be fit by a single common slope, representing the concentration dependence over all sgRNAs, regardless of sgRNA strength.

In this log-sigmoid transformed space, the concentration-dependence and effect of sgRNA strength have been decoupled (non-interacting), and thus are independent linear terms with the Hill coefficients ($H_{s}$ and $H_{d}$) as the variables to solve for by a standard regression. The inflection parameters of the sigmoid curve ($K_{s}$ and ${\text{EC}}_{50}$) are combined as the intercept C in the model. Importantly, this model implies that the effect of growth impairment due to the depletion of a vulnerable gene and growth inhibition due to the drug on the overall (relative) abundance of a given mutant are independent, because the effects are an “additive” in log-space. To illustrate this, the CRISPRi-DR equation is simulated by plotting idealized relative abundances (in Fig 2) using parameters chosen to emulate what is seen in Fig 1B; the *rpoB* plot of slopes over a systematic range of sgRNA strengths and drug concentrations. In Fig 2A, the slopes of the concentrations are plotted against abundances calculated using the dose-response model. The slopes change as a function of the starting depletion (left-hand side), which varies due to sgRNA strength alone (colored by blue-orange gradient based on strength value). The slopes are most negative for intermediate sgRNA strength, colored with a dark blue-green hue representing sgRNA strength (extrapolated LFCs) around −10. Fig 2B shows the result of the linearization of the Hill equation. All the individual sgRNA regression lines over concentration become parallel, eliminating the dependence on sgRNA strength, and allowing them to be fit by a single common slope representing the concentration-dependence averaged over all the sgRNAs.

The data (sgRNA relative abundances from sequencing) are fit on a gene-by-gene basis using ordinary least-square (OLS) regression by the following formula:

[5] $$\log\left(\frac{A_{ijk}}{1-A_{ijk}}\right)=\beta_{0}+\beta_{c}\cdot \log([D_{j}])+\beta_{s}\cdot S_{i}$$

where A (relative abundance for each sgRNA at given drug concentration), $S_{i}$ (sgRNA strength estimated by predicted log fold depletion at 25 generations based on passaging) and $\left[D_{j}\right]$ (concentration of drugs) are columns of a melted matrix. To include the control samples (no-drug ATC-induced controls, concentration 0) in the regression, they are treated as one two-fold dilution lower than the lowest available concentration tested for the drug (to avoid taking the log of 0). Since the log-sigmoid transform of the relative abundances is taken, they must be within the range of (0,1) but not equal to either extremum. While relative abundances are generally non-negative, they can be greater than 1.0, reflecting sgRNAs that increase in abundance with drug concentration relative to the uninduced (−ATC) condition. To account for this, the following squashing function is applied to adjust outlying values to be within the desired range, while retaining monotonicity:

[6] $$A_{ijk}=\tau +\frac{\left(1-\tau )(1-e^{-2A_{ijk}}\right)}{\left(1+e^{-2A_{ijk}}\right)}$$

where τ=0.01 is a pseudo count needed to make abundances non-zero for taking logarithms.

### Significance Testing

**Fig 3.**
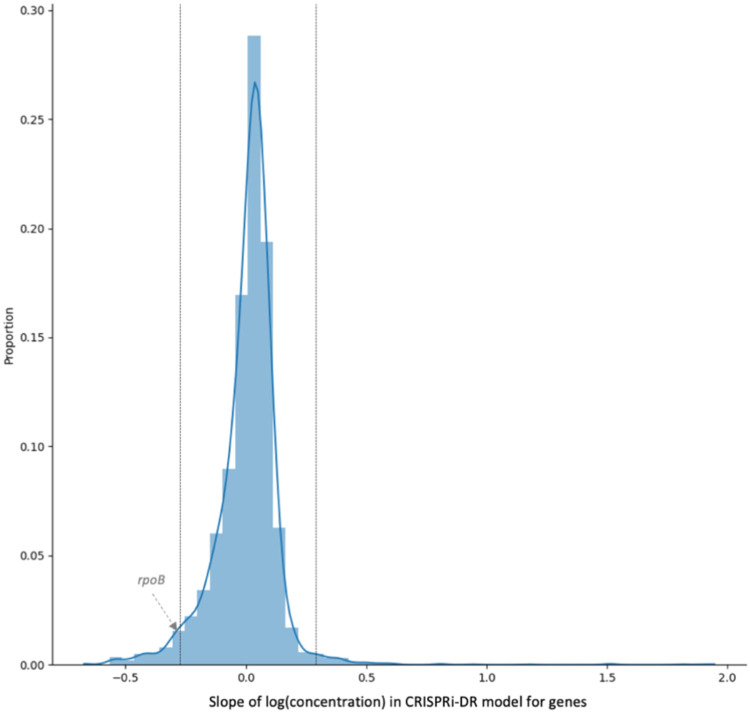
Coefficient of log-dependence from CRISPRi-DR model fitted for RIF D1 (1 day of pre-depletion). The distribution of the slopes of concentration dependence, extracted from the model fit for each gene. The vertical lines are at slope = −0.28 and slope = 0.28. These are the slopes adjusted p-value < 0.05 and the ∣Z-score∣> 2.0. 195 genes have significant slope values, i.e., 195 genes show a significant change in abundance with increasing RIF concentration while accounting for sgRNA strength. *rpoB* is significant with a slope of −0.29.

The statistic that indicates the degree of interaction of each gene with a given drug is the coefficient for the log([D]) term (i.e. slope) in the model. To determine whether the interaction is statistically significant, a Wald test [7] is applied to calculate a p-value reflecting whether the coefficient is significantly different than 0, adjusting for a target FDR (false discovery rate) of 5% over the whole genome using the Benjamini-Hochberg procedure [8]. However, the Wald test by itself yields too many hits (i.e., the genes predicted to have the greatest interaction with the drug, with adjusted p-value < 0.05). The test selects genes with slopes that are technically different than 0, but not necessarily large enough to be biologically meaningful. Therefore, genes are filtered based on the magnitude of the slopes, analogous to the criterion of ∣LFC∣>1, used by Li et al. [2], to filter significant genes by MAGeCK. The distribution of slopes over all genes is assumed to be a normal distribution, and the Z-scores are computed for every gene g: $Z_{g}=\frac{\beta_{c,g}-\mu (\beta_{c})}{\sigma (\beta_{c})}$ , where $\sigma (\beta_{c})$ is the standard deviation of the slopes of log concentration dependence and $\mu (\beta_{c})$ is the mean of the slopes. Genes with $|Z_{g}|<2.0$ are filtered out. This produces hits whose slopes are significant outliers (>2σ)from the rest of the population (genes in the genome). There are two groups of hits, corresponding to the two tails of the distribution: enriched hits where $Z_{g}>2.0$, and depleted hits, $Z_{g}<-2.0$. Fig 3 shows the distribution of the slopes calculated for genes in a library treated with RIF (one day of pre-depletion, D1). The threshold for this distribution where $|Z_{g}|>2.0$ and adjusted p-value < 0.05, is at slope = −0.28 and slope = 0.28 (vertical bars). The 195 total genes in the tails outside the vertical lines are identified as significant genes. These genes include the target of RIF, *rpoB*.

## Results

### CRISPRi data and pre-processing

The data was obtained from high-throughput sequencing of a CRISPRi library of *M. tuberculosis* (*Mtb*) of 96,700 sgRNAs [2]. For all 4019 genes in the Mtb H37Rv genome, there is an average of 24 sgRNAs per gene (range: 4-711). This library was intentionally constructed to focus on probing essential genes (based on prior TnSeq analysis [9]), with a mean of 83 sgRNAs per essential gene but there are some sgRNAs in each non-essential gene (mean of 10 sgRNAs per non-essential gene).

Samples of the library induced with ATC, in the presence of a drug were sequenced in triplicate at several concentrations for each drug at 2-fold dilutions around the MIC, along with control samples representing the no-drug ATC-induced samples (0 concentration). Three periods of pre-depletion (+ATC, prior to antibiotic exposure) were evaluated: 1, 5, and 10 days (D1, D5, and D10). The measurements reported in this library are observed barcodes counts of mutants in a culture, each with a different sgRNA, representing the relative proportion of each mutant in the population (i.e., abundance). However, abundance can increase or decrease if a vulnerable gene is depleted through CRISPRi interference, causing a change in fitness. Although levels of a target protein are knocked down by transcription interference via CRISPRi, protein levels are not directly measured. The barcodes that are being counted are nucleotides amplified from plasmids in the cells. This indirectly reflects the growth defect caused by depletion of a vulnerable gene. Each individual sample consisted of a vector of 96,700 barcode counts. Samples were normalized by dividing individual counts for each sgRNA by the sample total (sum over all sgRNAs).

Prior estimates of sgRNA strengths are also required. These were obtained from empirical data by fitting a piecewise-linear equation to fitness over multiple generations, and then inferring the predicted log-fold change at 25 generations [1]. As the absolute effect of depletion solely due to the sgRNA induction plays an important role in the CRISPRi-DR model (below), the analysis also requires samples representing abundance of mutants in the absence of −ATC (no dCAS9 expression, and hence no depletion of target transcripts by sgRNAs).

### sgRNA strength shows a strong correlation with abundance

**Fig 4.**
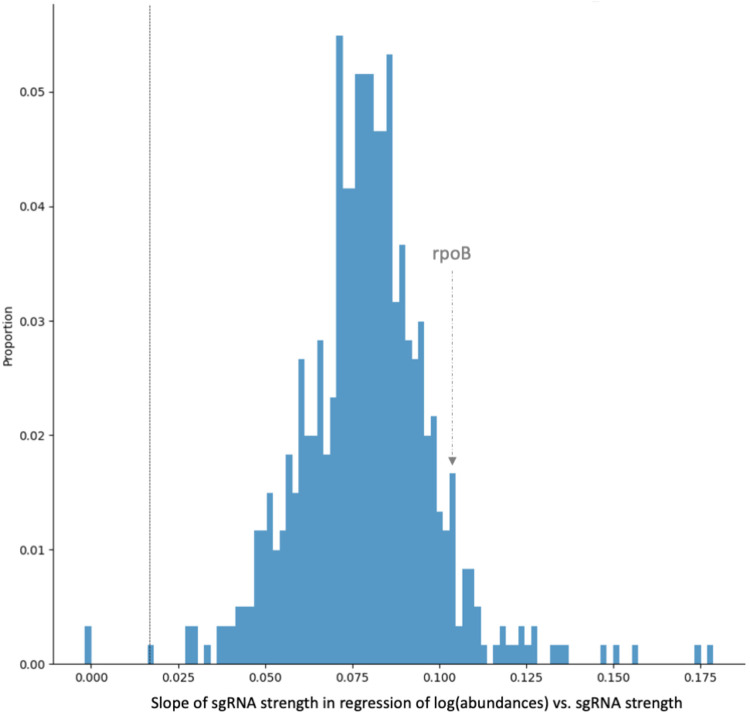
Distribution of slopes from regression of log_10_ (abundances) with respect to sgRNA strength, fit for the RIF D5 dataset. For essential genes in the RIF (D5) experiment with at least 20 sgRNAs, we regressed the average log normalized relative abundance at no-drug control samples against the sgRNA strengths (extrapolated LFCs at 25 generations) and plotted a histogram of the coefficients. sgRNAs that are significant are those with slope >= 0.024 (adjusted p-value < 0.05). Most of the slopes are greater than 0 and marked as significant. As sgRNA strength increases for a mutant, abundance decreases, indicating a direct relationship between sgRNA strength and mutant depletion.

sgRNA strength shows a linear trend with log (abundances) in essential genes. For example, Fig 1 illustrates a strong relationship between sgRNA strength and mutant growth suppression for *rpoB* (RNA polymerase). This can be quantified as the slope of the regression: $\log_{10}\;A_{ik}=B\cdot S_{i}+C$, where $A_{ik}$ is the relative log abundance of an sgRNA in replicate k (counts in +ATC culture divided by counts in - ATC), $S_{i}$ is the strength of sgRNA i in the form of extrapolated LFCs (calculated for the library grown in - ATC in buffer ), and C is the intercept. This regression was run on essential genes with at least 20 sgRNAs. Non-essential genes were excluded in this analysis since they have fewer sgRNAs in the library and tend not to deplete regardless of concentration or sgRNA strength. As seen in the distribution in Fig 4, most of genes show slope greater than 0 (though not all as large as *rpoB*), and nearly all are significant (Wald test, adjusted p-value < 0.05). In all the genes, as sgRNA strength increases (i.e. extrapolated LFCs become more negative), abundances decrease. This demonstrates that there is a direct relationship between sgRNA strength and mutant depletion extending to all essential genes in the genome. Therefore, strength of the sgRNAs is an important covariate of predicting abundances and should be incorporated in the model to accurately identify genes showing depletion in a condition.

### The CRISPRi-DR model accurately predicts sgRNA abundances from sgRNA strength and drug concentration

For all experiments, the CRISPRi-DR model with both sgRNA strength and concentration as predictors outperforms reduced models. When the model is run on each gene in the ethambutol (EMB D5) experiment, 59.2 % of the 4032 genes show $r^{2}$ values (correlation of predicted and observed abundances) of at least 0.5. As expected, these genes include targets of EMB, *embA*, *embB* and *embC* as well as other cell wall related genes such as the *aft* (arabinofuranosyltransferase) genes.

To evaluate the relative importance of the sgRNA strength and drug concentration features to the CRISPRi-DR model, each gene was run through two ablated models: M_d_ and M_s_. The M_d_ model contained only log concentration as a predictor: $\log\left(\frac{A_{ijk}}{1-A_{ijk}}\right)=B\cdot \log\left(\left[D_{j}\right]\right)+C$ and the M_s_ model only contained sgRNA strength as a predictor: $\log\left(\frac{A_{ijk}}{1-A_{ijk}}\right)=B\cdot S_{i}+C$. In the EMB D5 experiment, only 33.4% of genes fitted with M_s_ and 8.0% of genes fitted with M_d_ show $r^{2}$ values at least 0.5. *embA*, *embB* and *embC* do not appear in the either of these sets of significant interactors. The average log-likelihood (LL) of the full model in the EMB D5 experiment is −99.5, whereas the average log-likelihood of M_d_ is - 245.1 and average log-likelihood of M_s_ is −131.4 (higher LL values represent better fit). When the log-likelihood ratio (LR) test is performed, the LR-statistics show that M_s_ is an improvement over M_d_, and the full model is a greater improvement over both M_d_ than M_s_. In all three models, most of the insignificant genes (adjusted p-value of LR statistic ≥ 0.05) were non-essential genes that do show much depletion regardless of concentration or sgRNA strength. For targets of EMB, *embA*, *embB* and *embC*, the LR statistic for M_s_ is higher than M_d_ and is the highest in the full CRISPRi-DR model. The $r^{2}$ values and results of the log-likelihood ratio test indicate the sgRNA strength contributes more strongly to the CRISPRi-DR model than the drug concentration and is the dominant feature for most genes. Additionally, the full CRISPRi-DR model not only provides better fits for a greater quantity of genes than the ablated models, but it also provides betters fits for targets of the drug.

The CRISPRi-DR model’s improved performance over the reduced models for EMB extends to all drugs tested, as seen in S1 Fig. The dashed line in the plot indicates $r^{2}=0.5$. In all the experiments, the number of genes with fits that have $r^{2}>0.5$ is greater in the M_s_ model than M_d_. The number of genes with fits with $r^{2}>0.5$ is the greatest in the full CRISPRi-DR model. This demonstrates that in all conditions, both concentration and sgRNA strength are needed to make accurate estimates of sgRNA depletion.

Some users may not have the resources to run passaging experiments for all sgRNAs in their CRISPRi library to determine sgRNAs strengths empirically, and thus may want to rely on the predicted strengths based on sequence features. To evaluate how much of a difference the predicted strength in place of empirical strength, we fitted the CRISPRi-DR model on all the datasets with predicted strength in place of empirical strength and compared the results. The significant genes reported by the CRISPRi-DR model using predicted strength (based of sequence features) were nearly identical to the significant genes reported by the CRISPRi-DR model using empirical strength (based on passaging). The average overlap of interacting genes detected is 93.3%, with 24 out of 26 datasets having an overlap greater than 90%. Thus, using predicted sgRNA strengths is almost as good as using empirical estimates from passaging.

### CRISPRi-DR and MAGeCK have a high concordance of predicted gene-drug interactions

**Fig 5.**
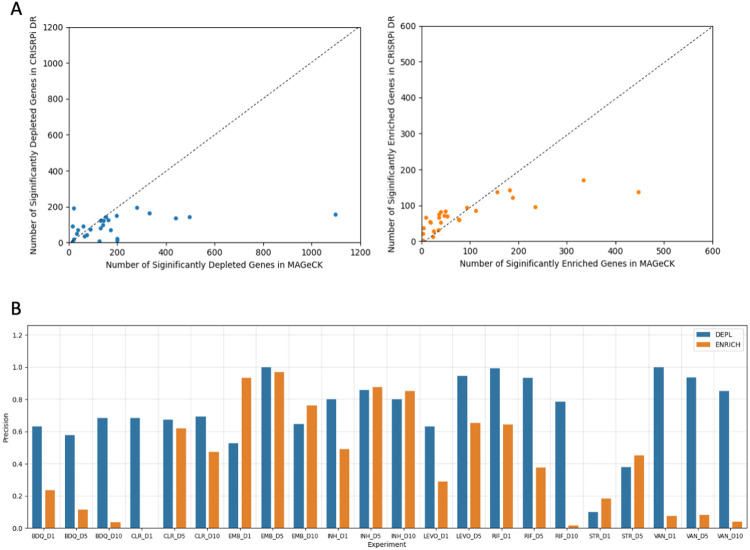
Comparison of significant interactions in CRISPRi-DR and MAGeCK. (A) The number of hits (both enriched and depleted) are slightly greater in MAGeCK than in the CRISPRi-DR model. However, both models produce comparable number of significant genes. The outlier point seen in for the scatterplot comparing depleted genes (top) is for VAN D1. The number of genes reported in the CRISPRi-DR model span a shorter range than the number of genes reported in MAGeCK. (B) Precision of significant genes reported by the CRISPRi-DR model. Overall, the precision of both enriched and depleted hits in the CRISPRi-DR model (compared to MAGeCK) are high. There is a greater overlap in depletion hits than enriched hits. The LEVO D10 and LZD datasets had almost no hits in MAGeCK [see Extended Data Fig 2 in (Li, Poulton et al. 2022)]. As a result, they were excluded from the precision analysis.

The overall number of significant genes identified by the CRISPRi-DR model is comparable to those reported by MAGeCK, but MAGeCK identifies additional genes that are not detected as significant by the CRISPRi-DR model. MAGeCK and CRISPRi-DR detect about the same number of significantly enriched and depleted genes, typically on the order of tens to a few hundred for any given drug, as shown in Fig 5A. The number of false negatives (significant in MAGeCK but not in CRISPRi-DR) are balanced with the number of false positives (significant in CRISPRi-DR but not in MAGeCK); they are both on similar scales. On average, 57.5% of significant genes in CRISPRi-DR are also significant genes in MAGeCK. However, for some drugs, MAGeCK predicts substantially more hits. For example, MAGeCK finds over 1066 significantly depleted genes for VAN (even with the filter of ∣LFC∣>1 applied), whereas CRISPRi-DR finds only 196 significant interactors.

**Fig 6.**
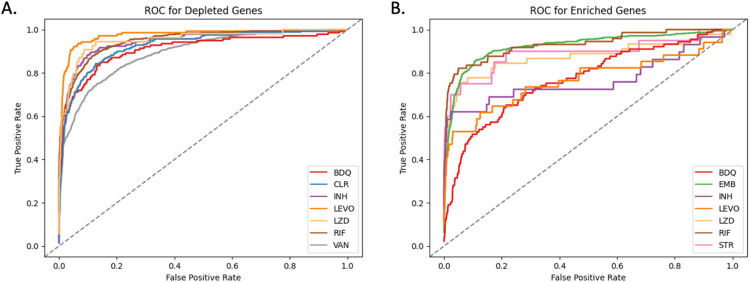
ROC curves comparing gene rankings in MAGeCK and CRISPRi-DR for enriched hits (A) and depleted hits (B) in 1 day pre-depletion experiments. The recovery of the depleted hits outperforms the recovery of enriched hits, showing that MAGeCK and the CRISPRi-DR model rank depleted genes similarly. EMB and STR are excluded in the ROC analysis of depleted genes and CLR and VAN are excluded in the analysis of enriched genes. These libraries had too few significant genes reported by MAGeCK in their respective categories to yield meaningful ROC curves. The lower performance of the enrichment gene rankings may be due to a few reasons, including noise.

The ranking of genes using the CRISPRi-DR model (using coefficient of concentration dependence, as described above) correlates well with ranking of genes in MAGeCK. For each of the 9 drugs tested, Receiver Operator Characteristic (ROC) curves were calculated for the D1 (1 day) pre-depletion datasets, seen in Fig 6. The average areas under curves (AUC) in Fig 6A is 0.95, indicating that the genes reported in MAGeCK across all concentrations are ranked highly in the CRISPRi-DR model. For instance, 70.0% of the top-100 ranked depletion genes in MAGeCK appear in the top-100 ranked depletion genes in the CRISPRi-DR model. The areas under the curves in Fig 6B for enriched hits are lower than of Fig 6A , with an average of 0.83.

The discrepancy between interactions detected by MAGeCK and CRISPRi-DR for enriched hits can be observed as an imbalance between false negatives and false positives in the confusion matrices (see S2 Table). Many genes with significant enrichment by MAGeCK are not called significant by CRISPRi-DR. This imbalance can be quantified as *precision* (calculated as TP/(TP+FP), or fraction of true positives (defined by MAGeCK) vs all positives (predicted by CRISPRi-DR). The precision of these CRISPRi-DR calls can be seen in Fig 5B. The average overlap of significantly depleted genes is 73.3%, whereas the average of significantly enriched genes is nearly half that, at 41.7%. The significant genes reported using the CRISPRi-DR model are largely a subset of the genes reported by MAGeCK, with a smaller overlap of significant enriched genes than significant depleted genes. This lower concordance of the two models for *enriched* hits shows that MAGeCK may be selecting genes with large variations, deceptively seeming to be significant interactions, that the CRISPRi-DR model does not. This might be attributable to the greater susceptibility of MAGeCK to noise in barcode counts, which is higher for some enriched genes (discussed below).

### CRISPRi-DR model correctly detects genes known to interact with anti-tubercular drugs.

When genes are ordered by coefficients of the slope representing the dependence of abundance on drug concentration from the CRISPRi-DR model, genes for existing anti-mycobacterial drugs are ranked highly, as expected (Table 1). The more positive a gene’s coefficient is, the higher the gene’s enrichment ranking, and the more negative a gene’s coefficient is, the higher it’s depletion ranking.

Genes that are known to be involved in the target mechanism of a drug should have a high depletion rank, i.e., show a negative slope, indicating that as concentration increases, abundance for the given depletion-mutant decreases. This can be seen in S1 Table, in the ranking for genes using the CRISPRi-DR model. *embA*, *embB*, and *embC* (subunits of the arabinosyltransferase, target of ethambutol, EMB) rank within the top 100 depleted genes for all three pre-depletion conditions for EMB. They rank the highest in D1 and the lowest in D10. This can be attributed to the fact that by D10 genes are already quite depleted, even at concentration 0, increasing noise, and making it difficult to pick up on depletion signals over increasing concentration. Therefore, the ranking of relevant genes in D1 was assessed in this analysis (Table 1). In RIF, target genes *rpoB*, *rpoC* are ranked within the top 150 genes. Significant negative interacting genes for RIF also include many cell wall related genes such as *ponA2*, *rodA*, *ripA*, *aftABCD*, *embABC*, etc., consistent with recent studies that show RIF exposure (or mutations in *rpoB*) leads to various cell wall phenotypes [10-12]. Similarly, the targets of bedaquiline (BDQ), the 8 ATP synthase genes (*atpA*-*atpH*, subunits of F0F1 ATP synthase), along with efflux pump *mmpL5*, are ranked within the top 40 depleted genes in BDQ. In levofloxacin (LEVO), *gyrA* and *gyrB* (subunits of the DNA gyrase, the target of fluoroquinolones) are observed to be enriched. The reason that depletion of this drug target leads to enrichment of mutants (hence a growth advantage, rather than the expected growth impairment) is likely due to reduced generation of double-stranded breaks in the DNA and other toxic intermediates as a side-effect of inhibiting the gyrase, an effect that has been observed in *E. coli* [13]. The significantly depleted genes in vancomycin (VAN) show significant enrichment for the cell wall/membrane/envelope biogenesis pathway (as defined by in COG pathways [14]) using Fischer’s Exact Test This follows previous studies that show cell wall genes are targets of vancomycin [15, 16], which binds to peptidoglycan in the cell wall. For clarithromycin (CLR), an inhibitor of translation, *Rv3579c* and *erm(37)* are observed as hits. *Erm(37)* adds a methyl group on the A2058/G2099 nucleotide in the 23S component of the ribosome, the same position to which CLR attempts to bind [17]. This natively increases tolerance to CLR in *Mtb*. As this gene is depleted, CLR has greater opportunity to bind, reducing the bacillus’ natural tolerance to the drug. Following this observation, *erm(37)* has a depletion rank of #1 in the CLR D1 condition. *Rv3579c* is another methyltransferase with a similar function that ranks highly (#35) in CLR.

In contrast to methylation inhibiting the binding of CLR, there are ribosome methyltransferases where methylation facilitates binding of a drug. Mutants for these genes would be expected to show a high enrichment rank in presence of drug. For instance, streptomycin (STR) interferes with ribosomal peptide/protein synthesis by binding near the interaction of the large and small subunits of the ribosome [18]. Two relevant genes that influence the binding of STR include *gidB* and *Rv2477c/ettA*. *gidB* is an rRNA methyltransferase that methylates the ribosome at nucleotide G518 of the 16S rRNA, the position at which STR interacts [19], increasing native affinity for STR. This is consistent with the observation that one of the most common mutations in STR-resistant clinical isolates is loss of function mutations in *gidB* [20]. *Rv2477c* is a ribosome accessory factor, also known as *ettA*, which is an ATPase that enhances translation efficiency. It has also recently been shown to bind the ribosome near the P-site (peptidyl transfer center), potentially interfering with binding of aminoglycosides [21], and loss-of-function mutations observed in drug-resistant clinical isolates of *M. tuberculosis* have shown to confer resistance to STR [2]. The ranking of both genes using the CRISPRi-DR model are within the top 12 enriched genes in STR. For linezolid (LZD), relevant genes identified are *erm(37)* and *tsnR*. *tsnR* is an rRNA methyltransferase, analogous to *gidB* and results in tolerance to LZD in a similar manner as *gidB* does for STR [2]. Following this expectation, *tsnR* has an enrichment ranking of #1 in LZD. Whereas depletion of *erm(37)* gives tolerance to CLR, it increases sensitivity to LZD. The nucleotides that *erm(37)* methylates in the 23S RNA are proximal in 3D space to where mutations conferring LZD-resistance are found, which both lie in the PTC (peptidyl-transfer center) of the ribosome [22].

For isoniazid (INH), there are multiple relevant genes identified by CRISRPi-DR, including *inhA*, *ahpC*, *ndh* [23], and *katG* [24]. *inhA* (enoyl-ACP reductase, in mycolic acid pathway) is an essential gene that is the target of INH, and *ahpC* (alkyl hydroperoxide reductase) responds to the oxidative effects of isonicotinic radicals in the cells. Therefore, as dosage of the drug increases, the abundances of the mutants of these genes should decrease. These genes are in the top 10 highest ranked depletion genes for INH (see Table 1). In contrast, *katG* and *ndh* are found among the top 5 enriched hits, exhibiting increased survival when the proteins are depleted. KatG (catalase) is the activator of INH, and the most common mutations in INH-resistant strains occur in *katG*, decreasing activity [25]. *Ndh* (type II NADH reductase) mutants have also been shown to decrease sensitivity to INH by shifting intracellular NADH levels (needed for INH-NADH adduct formation), and mutations in *ndh* have been shown to be defective in target enzyme (NdhII) activity [23], which is consistent with the observation in the CRISPRi data that depletion of *ndh* leads to increase survival in the presence of INH.

### The CRISPRi-DR model is less sensitive to noise than MAGeCK

MAGeCK’s greater sensitivity to noise could be a reason that the CRISPRi-DR model shows lower consistency with MAGeCK for enriched hits (e.g. lower AUC in Fig 6B than Fig 6A). There is some noise in these experiments due to variability in sequencing barcode counts across replicates. This can differentially affect the accuracy of predictions of gene-drug interaction made by these models. Three replicates were available for each measurement, i.e., 3 different counts estimating the relative abundance of each sgRNA in the presence of a drug at a given concentration. Coefficient of variation (CV) can be used to measure relative consistency across these observations for each measurement, which in turn can be used to evaluate MAGeCK and the CRISPRi-DR model’s sensitivity to noise in the raw data.

**Fig 7.**
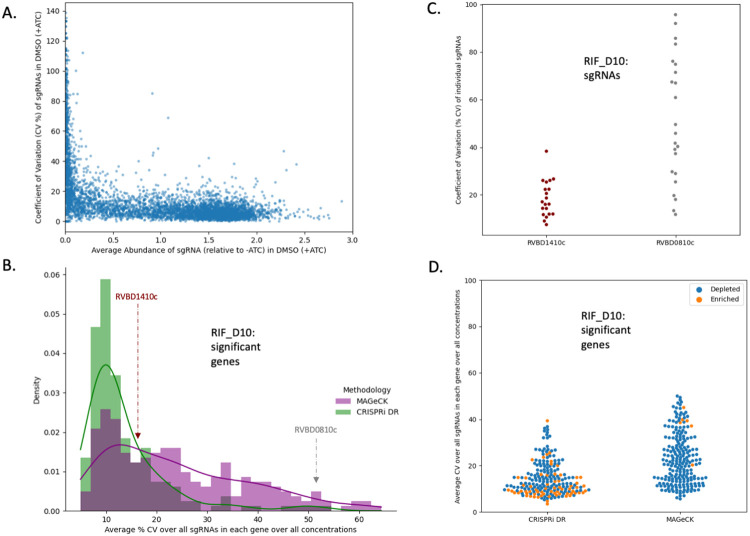
CRISPRi-DR model shows less sensitivity to noise than MAGeCK. (A) Comparison of average relative abundance and average CV across replicates in no-drug control samples (+ ATC) for a sample of sgRNAs : For each sgRNA, we looked at the average CV of sgRNAs in the 3 control replicates against the average abundance of the sgRNA across those replicates. The lower the average abundance, the greater the noise present for the sgRNA. (B) Distribution of average CV of gene for significant genes in MAGeCK and significant genes in CRISPRi-DR in RIF D10 : The distribution of average CV of significant genes in CRISPRi-DR model is more skewed and has a peak at CV ≈ 10%. Although most significant genes in MAGeCK show an average CV around 15%, there are quite a few genes with higher average CVs not found significant by the CRISPRi-DR model. (C) Coefficient of Variation (CV) of each sgRNA in two genes with similar number of sgRNAs for a library treated with RIF D10 : *Rv1410c* is significant in both methodologies and *Rv0810c* significant in MAGeCK but not in CRISPRi-DR. The majority of CV values for sgRNAs in *Rv1410c* is around 20%. Although both genes have about 20 sgRNAs, *Rv0810c* shows 8 sgRNAs whose CV values exceed 60.5%, which is the maximum CV present in *Rv1410c*. (D) Distribution of average CV for enriched and depleted significant genes in MAGeCK and CRISPRi-DR in a RIF D10 library. This plot shows the distribution plot of Panel B, separated by depletion and enriched significant genes. The average CV values for significant genes in the CRISPRi-DR model are low for both enriched and depleted genes. As seen in Panel B, significant genes in MAGeCK show low average CV, but they also show high average CV. Although there is a substantially lower number of significantly enriched in MAGeCK, they still show a large amount of noise compared the significantly enriched genes in CRISPRi-DR model.

For each sgRNA $s_{i}$ the coefficient of variation (CV) was calculated across the relative abundances for the 3 replicates for each concentration © in drug (D) ($CV_{D,C,i}=\frac{\sigma (i)}{\mu (i)}$), where σ(i) is the standard deviation of the 3 relative abundances in concentration C and μ(i) is the mean. In Fig 7A, the $CV_{D=DMSO,C=0,i}$ (CV of abundances in concentration 0) for a random subset of sgRNAs (~5%) in an ATC-induced no-drug condition is compared to the average abundance. For sgRNAs of medium to high abundance, the CV is fairly constant at approximately 10%. However, as the average abundance decreases (below relative abundance of 0.1), CV value increases substantially to 140%. If a gene contains multiple such sgRNAs with high CV values, then the variation may be misconstrued as a genetic interaction by a noise-susceptible methodology.

The average noise in a gene g for a given drug D can be quantified as the average $CV_{D,C,i}$, for all concentrations C and all sgRNAs in the gene ($\bar{CV_{D}}(g)$). Therefore, $\bar{CV_{D}}(g)$ reflects the measure of overall noise present in a gene in a drug D. The distribution of $\bar{CV_{D}}(g)$ in RIF D10 for the 215 total significant genes (enriched and depleted combined) in the CRISPRi-DR model and in 218 total significant genes (enriched and depleted combined over all concentrations) in MAGeCK can be seen in Fig 7B. The distributions for both methodologies share a mode at about $\bar{CV_{D}}(g)\approx 10\%$. The distribution of $\bar{CV_{D}}(g)$ for significant genes in MAGeCK has a fatter tail than the distribution of $\bar{CV_{D}}(g)$ for significant genes in the CRISPRi-DR model. This trend is seen not only in RIF D10, but across all the experiments conducted (See S2 Fig). This indicates that although MAGeCK is identifying genes with low noise (like the CRISPRi-DR model), it is also detecting many genes with high noise that the CRISPRi-DR model is not.

An example of such a gene is *Rv0810c*. The gene has 22 sgRNAs and has a $\bar{CV_{D}}(g)$ value (average CV over sgRNAs in a gene) of 51.4%, one of the highest measures in the RIF D10 experiment. In RIF D10, it is reported to be significantly depleted only in MAGeCK and not in the CRISPRi-DR model. The distributions of the CV values for each sgRNA are compared to those of *Rv1410c* in Fig 7C. *Rv1410c* has 20 sgRNAs, an $\bar{CV_{D}}(g)$ of 16.3% and is reported to be significantly depleted in both MAGeCK and the CRISPRi-DR model. Although both genes have some sgRNAs with low CVs (below 40%), *Rv0810c* shows 8 sgRNAs with CVs of at least 60.5%, which is the maximum CV of sgRNAs in *Rv1410c*. The CRISPRi-DR model considers the abundances at all concentrations, whereas MAGeCK compares each concentration to the baseline independently. Therefore, if sgRNAs have a high CV value at a particular concentration, they can be picked up as a significant genetic interaction by MAGeCK. The average relative abundance for the 3 replicates at concentration 0 for all sgRNAs in *Rv0810c* is 0.19, whereas the average relative abundance in *Rv1410c* for the same is 1.08. As Fig 7A shows, *Rv0810c* falls in the low abundance/high noise section of the graph, with an average sgRNA no-drug CV of 47.9%, whereas *Rv1410c* falls in the low noise section of the graph, with an average sgRNA no-drug CV of 11.2%. This demonstrates that MAGeCK reports genes such as *Rv0810c* with low abundances resulting in large $\bar{CV_{D}}(g)$ , which the CRISPRi-DR model does not, i.e., MAGeCK is more suspectable to noise than the CRISPRi-DR model.

Furthermore, the $\bar{CV_{D}}(g)$ for significantly enriched genes in MAGeCK is higher than the $\bar{CV_{D}}(g)$ for significantly depleted genes. As seen in Fig 7B, both methodologies detect genes with $\bar{CV_{D}}(g)\approx 10\%$ in RIF D10. The $\bar{CV_{D}}(g)$ values for both significantly depleted and enriched genes in the CRISPRi-DR model are close to this value (Fig 7D). MAGeCK detects significantly depleted genes at around this value, but also detects genes with much larger $\bar{CV_{D}}(g)$ values. Although there are fewer significantly enriched genes reported in MAGeCK than CRISPRi-DR, they show a larger amount of noise compared the significantly enriched genes detected by CRISPRi-DR. Since the significantly enriched genes in MAGeCK show higher noise than either significantly enriched or significantly depleted genes in the CRISPRi-DR model, it might partially explain the lower levels of overlap (AUC) seen in the ROC curves for enriched genes in Fig 6B.

### Simulation

The sensitivity and accuracy of the CRISPRi-DR model and MAGeCK was assessed under different sources of noise using simulated barcode counts sampled from the negative binomial distribution [26], with means at different concentrations determined by the dose-response model (Eqn. 3). sgRNAs and their empirical strength estimates from a previous study [2] were used to simulate the combined effects of CRISPRi depletion and exposure to a virtual inhibitor at four concentrations (1uM, 2uM, 4uM, and 8uM), with three replicates each. The aim was to determine how noise within and between concentrations affects the performance of each method. Detailed information on the simulation is provided in the S1 File.

Four datasets (LL, LH, HL, and HH) were simulated by varying two noise parameters: $\sigma_{B}$ (variability of abundances between concentrations) and p (variability of replicates within a concentration, parameter of the negative binomial distribution). 50 genes were randomly selected for negative interactions (consistent depletion effects) and another set of 50 genes for positive interactions (positive biased trend). The negative interactions were simulated using the dose-response formula (Eqn. 3) above, whereas the positive interactions and non-interacting sgRNAs were simulated using small random slopes to reflect concentration dependent effects. CRISPRi-DR and MAGeCK were run ten times each on these 4 scenarios. MAGeCK was run independently for each drug concentration (2uM, 4uM, 8uM, compared to a no-drug control), while CRISPRi-DR was performed on all four concentrations simultaneously.

Both methods displayed high recall in the LL scenario (lowest noise) (CRISPRi-DR : 95.4%, MAGeCK : 84.6%) but their recall rates are slightly degraded in the HH scenario (highest noise) (CRISPRi-DR : 59.7%, MAGeCK : 70.5%). The difference in sensitivity to noise is more apparent in the *precision* of the two methods. In the HH scenario, MAGeCK generates nearly four times as many false-positive predictions (463.3), leading to a very low precision of approximately 13.3%, whereas CRISPRi-DR’s precision is 36.5%, with 104.2 false positives. This indicates that MAGeCK is prone to classifying non-interacting genes as hits when noise is high, likely due to stochastic count fluctuations at individual drug concentrations that may not be observed at other concentrations. Comparatively, CRISPRi-DR relies more on consistent trends in abundance across concentrations, and thus makes less erroneous false positive predictions. Notably, the consistent trends in abundance detected by this regression-based model are not required to change perfectly linearly with increasing log_2_ drug concentration. Rather, as long as, there is a general trend (increasing or decreasing) across concentrations, then the gene’s slope coefficient (concentration dependence) can still be significant. For example, abundances for some sgRNAs may drop off sharply at either end of the concentration range. Several examples of sgRNAs with these patterns are shown in S1 File.

To assess the impact of profiling a CRISPRi library at multiple concentrations on the performance of CRISPRi-DR and MAGeCK, we conducted the simulation above with high-noise settings (HH) and varying numbers of drug concentrations (1, 2, or 3) for 10 iterations each. The recall of both methods held fairly constant as concentrations were added. However, increasing the number of concentration points caused a significant decrease in the precision of MAGeCK from 21.2% to 13.2%. While MAGeCK shows susceptibility to false positives when evaluating only a single concentration point, this effect was amplified with more concentrations. This accumulation of errors explains the decrease in precision with additional concentration points. In contrast, CRISPRi-DR is more robust with respect to false-positive errors. By incorporating data from all available concentrations and identifying significant trends, CRISPRi-DR maintained higher precision that did not diminish with the addition of more concentration points.

## Discussion

CRISPRi can be used to conduct CGI experiments through several approaches. One approach is to modulate expression of dCAS9 (with an active nuclease function) to control expression of the target gene at various levels. This allows for the quantification of phenotype (e.g. growth rate in presence of inhibitor) as a function of expression level of a target gene. Typically, sgRNAs are selected that are validated to strongly bind their target genes and provide strong depletion [3]. Another strategy to generate mutants with graded phenotypes is by using parent sgRNAs that are progressively weakened through mutations [27]. Mutants with knock-down of a particular gene that exhibit a statistically significant depletion-dependent shift in MIC are deemed interactions. Alternatively, one can use a catalytically-dead dCAS9 (since binding to gene targets is sufficient to block transcription), and rely instead on a range of sgRNAs with varying strength (which can be barcoded separately and quantified independently) to evaluate depletion-dependent fitness effects [1]. In these CRISPRi libraries, stronger sgRNAs better inhibit expression of targets genes and cause greater protein depletion, which can better reveal interactions with drug treatment (through synergies). Inclusion of multiple sgRNAs with different strengths for each target gene can be used to test for expression-dependent sensitization to inhibitors.

The availability of CRISPRi data for multiple sgRNAs of different strengths for each target gene presents new challenges for statistical analysis for CGI experiments. In previous work [5], we showed that regressing the relative abundances of mutants in hypomorph libraries over concentrations (on log-scale) can be used to improve detection of CGIs. This regression approach captured dose-dependent behavior, i.e. genes whose decreased expression caused either suppressed or enhanced fitness that increases in magnitude with drug concentration (i.e. exhibits a trend, which is important for statistical robustness). The CRISPRi-DR method described in this paper extends this previous work by showing how effects of both drug concentration and sgRNA strength can be accommodated in the same model. What we are looking for, ideally, is genes that exhibit synergistic behavior with a drug, where depletion of a target protein induces excess depletion (or enrichment) of the mutants grown in the presence of an inhibitor, and this effect is concentration-dependent (exhibits dose-response behavior).

In theory, both CRISPRi depletion of essential genes and exposure to antibiotics should impair growth of CRISPRi mutants (at least for depletion of essential genes). One might expect to observe a depletion effect due to either increasing sgRNA strength, or drug concentration, each producing regression "slopes" (in log-transformed space), with slopes for sgRNAs targeting non-essential genes being expected to be flat, regardless of sgRNA strength. However, we observed that sgRNA strength and concentration effects are not independent - they interact in a non-linear way. sgRNAs that are too weak do not produce enough depletion of a drug target to cause sensitization (MIC shift), and sgRNAs that are too strong deplete a mutant to such low abundances that concentration-dependent effects are difficult to quantify. Often, there is a "sweet spot", or an intermediate sgRNA strength which maximizes the concentration-dependent effect (which could be different for each gene). Mathis et al. [27] suggested that dose-response behavior could be modeled with a classic Hill equation, where the number of mutations between the sgRNA sequence and target gene was used as a proxy for strength in a logistic function fitted to growth rate. However, this covariate was not explicitly combined with environmental variables (such as drug concentration) in their model. Our CRISPRi-DR model incorporates both sgRNA strength and drug concentration as parameters, and reproduces the non-linear interaction between them, where the "slopes" for the effect of drug concentration on relative abundance of mutants can be larger in magnitude for sgRNAs of intermediate strength, while being flatter (slopes closer to 0) for sgRNAs of high or low strength.

The strength with which different sgRNAs cause a growth phenotype depends on various factors affecting how well they bind to and suppress transcription of their genomic targets. First, the strength depends on how well the guide RNA matches the optimal PAM sequence, in order to be recognized by and recruit the dCAS9 nuclease [6]. Second, it depends on the length (typically 17-24 bp) and GC content of the complementary region that hybridizes with the chromosome. These sequence factors can be combined to make a predictive model of the effect on expression of target proteins, which has been shown to predict sgRNA strength with moderate accuracy (R^2^=0.74) (see Fig 2C in [1]). For greater accuracy, sgRNA strength can also be empirically quantified by conducting a passaging experiment. By inducing expression of the dCAS9 and measuring growth-rate over several generations, the strength of each sgRNA can be fit using a piecewise linear model and extrapolated to an implied depletion at a constant number of generations (e.g. estimated log2-fold-change of abundance in +ATC vs −ATC at 25 generations) [1]. However, for some labs that might prefer to use predicted strengths instead of running passaging experiments, we showed that using predicted strengths from sequence features with CRISPRi-DR in place of empirical strength produces results that are nearly as good.

In this paper, we showed that this non-linear interaction between sgRNA strength and drug concentration can be modeled using an augmented Dose-Response equation, in which terms for both effects are included. By fitting the parameters in this equation to CRISPRi data from a CGI experiment (normalized barcode counts), one can estimate the degree to which depletion of a given gene sensitizes cells to an inhibitor, and thereby identify CGIs. While various computational methods exist for fitting non-linear equations, such as the Levenberg–Marquardt algorithm [28], we chose to linearize the modified Hill equation by applying a log-sigmoid transform. The transformation enables us to express the equation in a linear form, where the parameters (EC_50_, Hill slopes, etc.) appear as coefficients of linear terms or constants. Consequently, we can use ordinary least-squares regression (OLS) to fit the model to the CRISPRi dataset.

An alternative approach for analyzing CRISPRi data is MAGeCK, which is a based on the DeSeq2 method for analyzing RNA-seq data [29]. It calculates LFCs for each sgRNA at each individual drug concentration and combines them using RRA (robust rank aggregation) to identify significant CGIs. When MAGeCK was developed, exploiting the spectrum of sgRNA strengths was not anticipated, so the sgRNAs in a gene are not treated differentially, and the RRA relies on the expectation that at least a subset of sgRNAs will be strong enough to elicit suppression of the target gene and produce a consistent effect on fitness (enrichment or depletion of mutant abundance), which will be detected as a signal through rank aggregation, i.e. several sgRNAs for a gene having exceptionally high or low LFCs.

In principle, one could imagine incorporating the number of days of pre-depletion into the regression approach of CRISPRi-DR. It is often observed that a longer pre-depletion period increases the sensitivity of the experiment and synergy with drug. However, we elected to treat the days of pre-depletion independently, to facilitate the comparison with the analysis in Li, et al [2]. In retrospect, a single day of pre-depletion (D1) has proven adequate for detecting known interactions in most CGI experiments conducted thus far. MAGeCK-MLE is an extension of MAGeCK that can incorporate additional covariates such as days of pre-depletion into the generalized linear model [30]. However, the maximum likelihood parameter estimation process used by MAGeCK-MLE can be time-consuming. CRISPRi-DR provides several advantages over MAGeCK. First, it explicitly incorporates sgRNA strengths as a covariate in the model, taking advantage of this useful information. Second, CRISPRi-DR integrates data over multiple concentrations via regression. This provides enhanced statistical robustness. In contrast, MAGeCK analyzes each drug concentration independently, comparing them to a no-drug control to compute LFCs. But with any single concentration point, there is a risk of detecting false positives (due to noise), which could cause spurious fluctuations in barcode counts, making LFCs possibly appear significant. The susceptibility to noise was evident in the experimental data as predictions made by CRISPRi-DR differed from MAGeCK more on datasets with higher coefficients of variation (S2 Fig). Ideally, it is better to collect data over multiple concentrations for CGI experiments, because it is difficult to know ahead of time what concentration will be optimal to test for each drug. While choosing the MIC for single-point assays might sound reasonable, the actual potency in the CRISPRi experiment could shift due to expression of the dCAS9, inoculation effects, etc. Hence, CGI data is usually collected over a range of concentrations, with the hope that one or more of them will be near the inhibition-transition point. Furthermore, it is not always the case that the highest concentration should be the most informative one for detecting CGIs, as it might cause too much growth inhibition, making it difficult to assess dose-dependent behavior.

A simplistic way to use MAGeCK with CGI data collected over multiple drug concentrations is to evaluate each concentration independently, and then combine selected hits (significant genes) using a policy such as taking the union [2]. However, our simulation results showed that this strategy is susceptible to accumulating false positive hits (i.e. non-interacting genes that achieve statistical significance), resulting in low precision. In fact, in previous experiments with a CRISPRi library in *Mtb*, MAGeCK often identified hundreds of genes (and in some cases, up to one-quarter of the genome) as potential interactions for certain antibiotics. While it is true that a variety of genes could interact with a drug directly or indirectly (not just the drug target), revealing multiple complex drug-tolerance and stress-response pathways, it is implausible that there will be hundreds of genuine interactions for most inhibitors. The CRISPRi-DR approach addresses this issue by requiring that apparent interactions (depletion or enrichment) at one concentration be consistent with trends in abundance at other concentrations. The abundance does not have to change in a perfectly linear way over the concentration range (which is helpful, because sometimes the largest effect occurs at the edge of the range, like dropping off a cliff, due to uncertainty about the optimal concentration), but large fluctuations in abundance in the middle of the range, or in opposite directions at different concentrations, will generally get filtered out as insignificant by CRISPRi-DR. Thus, incorporating data from sgRNAs of different strength over multiple concentrations via the modified Dose-Response model make CRISPRi-DR more noise-tolerant and robust for detecting chemical-genetic interactions.

## Supplementary Material

Supplement 1

## Figures and Tables

**Table 1 :** Ranking of Select Genes using the CRISPRi-DR model in 1 Day pre-depletion of treated libraries.

Drug	Gene	D1 Depletion Ranking	D1 Enrichment Ranking
BDQ	*atpA*	11	4022
BDQ	*atpB*	6	4027
BDQ	*atpC*	35	3998
BDQ	*atpD*	12	4021
BDQ	*atpE*	23	4010
BDQ	*atpF*	7	4026
BDQ	*atpG*	9	4024
BDQ	*atpH*	8	4025
BDQ	*mmpL5*	2	4031
CLR	*RVBD3579c*	35	3998
CLR	*erm(37)*	1	4032
INH	*inhA*	6	4027
INH	*ahpC*	2	4031
INH	*katG*	4031	2
INH	*ndh*	4029	4
EMB	*embA*	4	4029
EMB	*embB*	5	4028
EMB	*embC*	12	4021
LEVO	*gyrA*	3834	199
LEVO	*gyrB*	3967	66
LZD	*erm(37)*	3994	39
LZD	*tsnR*	4032	1
RIF	*rpoB*	108	3925
RIF	*rpoC*	148	3885
STR	*ettA*	4023	10
STR	*gidB*	4022	11

For each drug, the CRISPRi-DR model is run on each gene (using data from D1). The coefficient for the slope of concentration dependence ($\beta_{c}$) is extracted from the fitted regression and used to rank the genes in both increasing order (for depletion) and inversely (for enrichment). Green reflects results consistent with expectations based on knowledge of known gene-drug interactions
