# Supplementary material for: A dose-response model for statistical analysis of chemical genetic interactions in CRISPRi screens
Source: bioRxiv. 2024 Feb 7:2023.08.03.551759. Originally published 2023 Aug 5. Preprint. [Version 2] doi: 10.1101/2023.08.03.551759 (PMC10418283; doi:10.1101/2023.08.03.551759)
Supplement: Supplement 3 [file NIHPP2023.08.03.551759v2-supplement-3.pdf]

bioRxiv preprint doi: https://doi.org/10.1101/2023.08.03.551759; this version posted February 7, 2024. The copyright holder for this preprint (which was not certified by peer review) is the author/funder, who has granted bioRxiv a license to display the preprint in perpetuity. It is made available under aCC-BY 4.0 International license.

Supporting Information

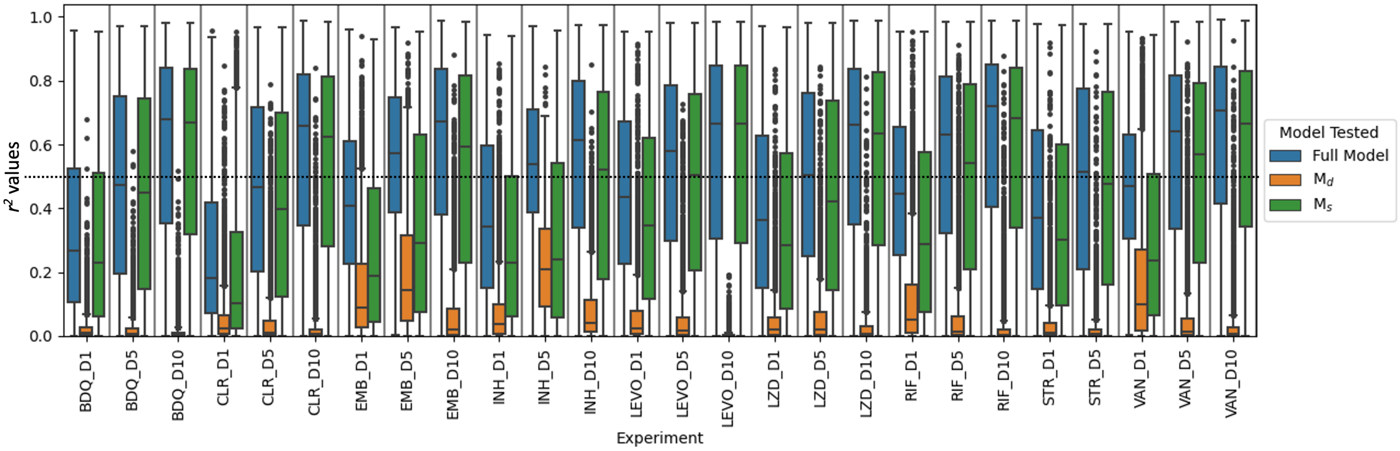

Fig S1 Evaluation sgRNA strength and log concentration as predictors of CRISPRi-DR model through comparison of distribution of r^2 values of full (CRISPRi-DR) and ablated (M_s and M_d) models for each gene in each experiment.

The horizontal line is where r^2 = 0.5. The average r^2 M_s model for all genes across all the experiments is 0.42, the average r^2 for the M_d model is 0.07. This alongside the Log-likelihood tests indicate sgRNA strength is the more significant predictor. However, the full CRISPRi-DR

bioRxiv preprint doi: https://doi.org/10.1101/2023.08.03.551759; this version posted February 7, 2024. The copyright holder for this preprint (which was not certified by peer review) is the author/funder, who has granted bioRxiv a license to display the preprint in perpetuity. It is made available under aCC-BY 4.0 International license.

model outperforms both M_d and M_s (average r^2 is 0.50) indicating the inclusion of both sgRNA strength and log concentration is needed for accurate assessment of significant sgRNA depletion in a gene in a condition.

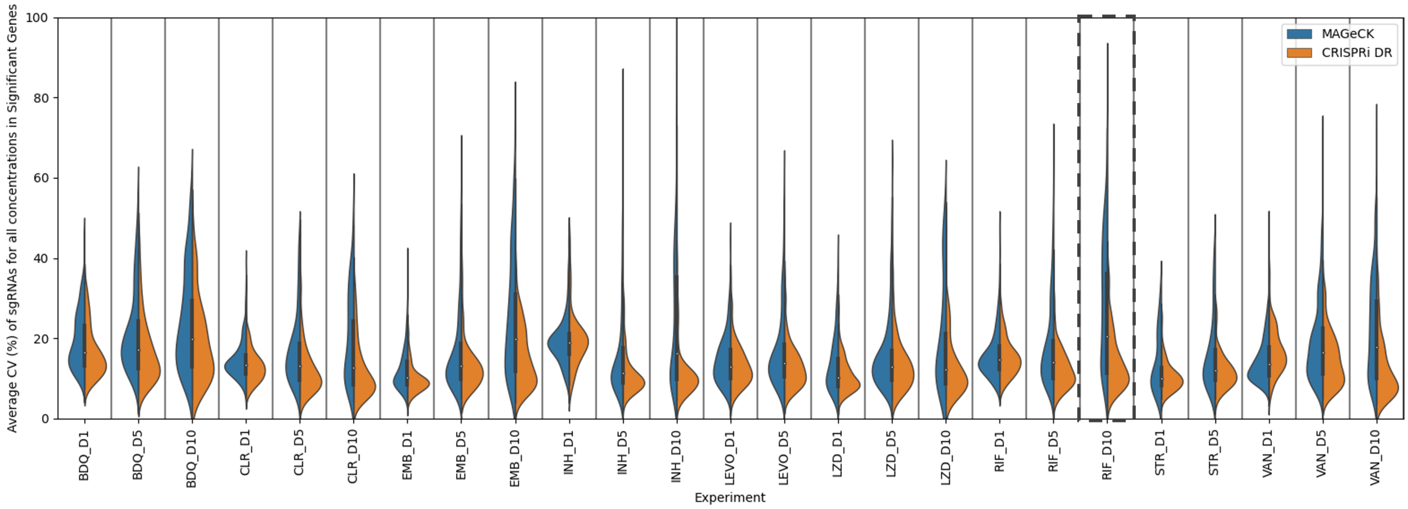

Fig S2 Distribution of average CV of sgRNAs in significant genes (depleted and enriched) in the CRISPRi-DR model and MAGeCK.

In this Fig, we see all the noise distributions for hits in MAGeCK and the CRISPRi-DR model for all experiments. The dashed panel is that of RIF D10. The same distribution of noise of hits can be seen in Fig 5. The trend seen with RIF D10 is present with all the experiments except LEVO D10. We see that the CRISPRi-DR model is unimodal with a low CV as the mode, whereas MAGeCK shows significant genes with low average CV values but also a significant amount of genes with high average CV values. LEVO D10 was left out of this plot due to the low number of hits in either model.

bioRxiv preprint doi: https://doi.org/10.1101/2023.08.03.551759; this version posted February 7, 2024. The copyright holder for this preprint (which was not certified by peer review) is the author/funder, who has granted bioRxiv a license to display the preprint in perpetuity. It is made available under aCC-BY 4.0 International license.

Table S1. Ranking of Select Genes using the CRISPRi-DR model in 1 Day, 5 day and 10 Day pre-depletion of treated libraries.

An extended version of Table 2, where the CRISPRi-DR model is run on each gene for each drug and pre-depletion day. The coefficient for the slope of concentration dependence (β_c) is extracted from the fitted regressions and used to rank the genes in both increasing order (for depletion) and inversely (for enrichment). Green reflects results consistent with expectations based on knowledge of known gene-drug interactions.

Table S2. Comparison of significant interactions Identified by CRISPR analysis methods of EMB, INH, LEVO, VAN and RIF CRISPRi screens

For each drug and pre-depletion day of the selected datasets, all 7 CRIPSR methods were run. For methods that do not account for multiple concentrations, they were run separately for each concentration and the overall significant interactions are also addressed post-combination of the individual runs using Fisher's method. The comparison of the significant interactions identified by the models was evaluated using an objectively defined list of true positives. The genes identified by Xu, DeJesus (35) were used as the "ground truth" against which the other model's results were compared. For LEVO, genes in the DNA Damaging pathway are used. Recall, Precision and F1-score columns are colored such that higher values are more green.

Table S3. Matrices for comparison of significant interactions Identified by CRISPRi-DR and MAGeCK for each drug and pre-depletion day.

bioRxiv preprint doi: https://doi.org/10.1101/2023.08.03.551759; this version posted February 7, 2024. The copyright holder for this preprint (which was not certified by peer review) is the author/funder, who has granted bioRxiv a license to display the preprint in perpetuity. It is made available under aCC-BY 4.0 International license.

The table presents the results of CRISPRi-DR and MAGeCK analyses for different drugs and pre-depletion days. Significant interactions are compared in matrix form. Cells with red font indicate low overlaps between the interactions found by the two models, while cells with green font represent high overlaps.

Supplemental File S1

We expand on the following four topics from the main text in this document: 1) An assessment of CRISPRi-DR, MAGeCK and MAGeCK-MLE on datasets with simulated noise, 2) Comparison of CRISPRi-DR to other analysis methods using CGI datasets, 3) Analysis of *E. coli* CRISPRi screens using CRISPRi-DR and, 4) The minimum number of sgRNAs recommended per gene in CRISPRi-DR.
