# Supplementary material for: A dose-response based model for statistical analysis of chemical genetic interactions in CRISPRi libraries
Source: bioRxiv. 2023 Aug 5:2023.08.03.551759. Preprint. [Version 1] doi: 10.1101/2023.08.03.551759 (PMC10418283; doi:10.1101/2023.08.03.551759)
Supplement: Supplementary file 1 [file NIHPP2023.08.03.551759v1-supplement-1.pdf]

bioRxiv preprint doi: https://doi.org/10.1101/2023.08.03.551759; this version posted August 5, 2023. The copyright holder for this preprint (which was not certified by peer review) is the author/funder, who has granted bioRxiv a license to display the preprint in perpetuity. It is made available under aCC-BY 4.0 International license.

Supporting Information

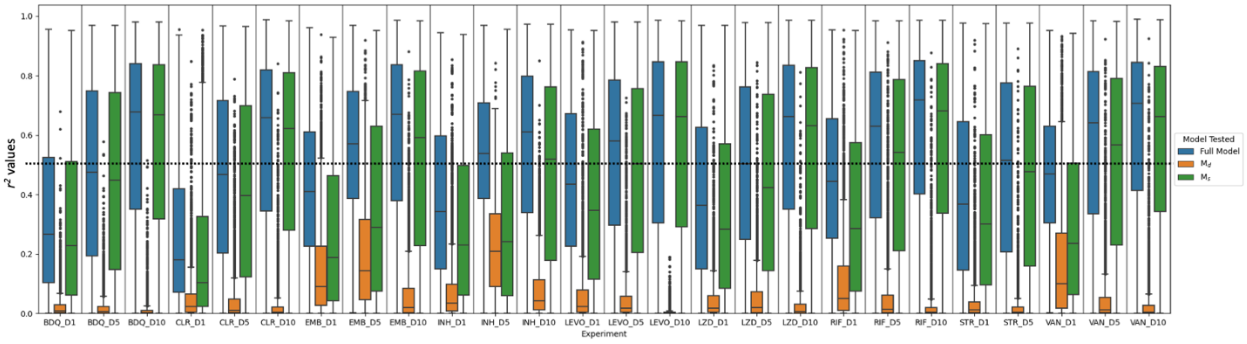

S1 Fig. Evaluation sgRNA strength and log concentration as predictors of CRISPRi-DR model through comparison of distribution of r^2 values of full (CRISPRi-DR) and ablated (M_s and M_d) models for each gene in each experiment.

The horizontal line is where r^2 = 0.5. The average r^2 M_s model for all genes across all the experiments is 0.42, the average r^2 for the M_d model is 0.07. This alongside the Log-likelihood tests indicate sgRNA strength is the more significant predictor. However, the full CRISPRi-DR model outperforms both M_d and M_s (average r^2 is 0.50) indicating the inclusion of both sgRNA strength and log concentration is needed for accurate assessment of significant sgRNA depletion in a gene in a condition.

bioRxiv preprint doi: https://doi.org/10.1101/2023.08.03.551759; this version posted August 5, 2023. The copyright holder for this preprint (which was not certified by peer review) is the author/funder, who has granted bioRxiv a license to display the preprint in perpetuity. It is made available under aCC-BY 4.0 International license.

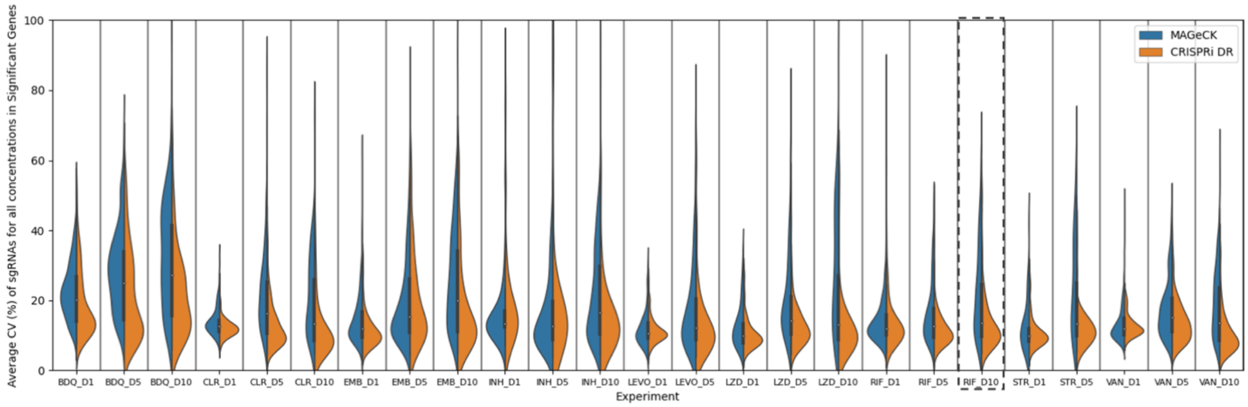

S2 Fig. Distribution of average CV of sgRNAs in significant genes (depleted and enriched) in the CRISPRi-DR model and MAGeCK.

In this Fig, we see all the noise distributions for hits in MAGeCK and the CRISPRi-DR model for all experiments. The dashed panel is that of RIF D10. The same distribution of noise of hits can be seen in Fig 7. The trend seen with RIF D10 is present with all the experiments except LEVO D10. We see that the CRISPRi-DR model is unimodal with a low CV as the mode, whereas MAGeCK shows significant genes with low average CV values but also a significant amount of genes with high average CV values. LEVO D10 was left out of this plot due to the low number of hits in either model.

S1 Table. Ranking of Select Genes using the CRISPRi-DR model in 1 Day, 5 day and 10 Day pre-depletion of treated libraries.

An extended version of Table 1, where the CRISPRi-DR model is run on each gene for each drug and pre-depletion day. The coefficient for the slope of concentration dependence (β_c) is extracted from the fitted regressions and used to rank the genes in both increasing order (for depletion) and inversely (for enrichment). Green reflects results consistent with expectations based on knowledge of known gene-drug interactions.

bioRxiv preprint doi: https://doi.org/10.1101/2023.08.03.551759; this version posted August 5, 2023. The copyright holder for this preprint (which was not certified by peer review) is the author/funder, who has granted bioRxiv a license to display the preprint in perpetuity. It is made available under aCC-BY 4.0 International license.

S2 Table. Comparison of significant interactions Identified by CRISPRi-DR and MAGeCK for each drug and pre-depletion day.

For each drug and pre-depletion day, both CRISPRi-DR and MAGeCK are run on data. MAGeCK is run separately for each concentration and the overall significant interactions are determined as the union of the individual runs. CRISPRi-DR is run is run once using data from all three concentrations (and sgRNA strengths) together. The comparison of the significant interactions identified by the models is evaluated using true positives, true negatives, false positives and false negatives. The results from MAGeCK are used as the "ground truth" against which the other model's results are compared. Cells with red font in the "tp" column represent low overlaps between the interactions found by the two models, and cell with red font in the "Number of …" columns highlight low number of interactions found in the relative model.

S3 Table. Matrices for comparison of significant interactions Identified by CRISPRi-DR and MAGeCK for each drug and pre-depletion day.

The table presents the results of CRISPRi-DR and MAGeCK analyses for different drugs and pre-depletion days. Significant interactions are compared in matrix form. Cells with red font indicate low overlaps between the interactions found by the two models, while cells with green font represent high overlaps.

S1 File. Evaluating performance differences between CRISPRi-DR and MAGeCK using a simulated sgRNA barcodes.

To better understand the differences in performance between CRISPRi-DR and MAGeCK, and to evaluate the sensitivity of these methods to different sources of noise, we developed a simulation model to generate artificial datasets of sgRNA barcode counts. In this experiment, we used the same set of ~99,000 sgRNAs and empirical measurements of sgRNA strengths for genes in the *Mtb* genome as in the

bioRxiv preprint doi: https://doi.org/10.1101/2023.08.03.551759; this version posted August 5, 2023. The copyright holder for this preprint (which was not certified by peer review) is the author/funder, who has granted bioRxiv a license to display the preprint in perpetuity. It is made available under aCC-BY 4.0 International license.

CRISPRi library in the paper by (Li, Poulton et al. 2022), and simulated exposure to a virtual inhibitor over 4 concentrations (1μM, 2μM, 4μM, and 8μM), 3 replicates each. Our objective was to quantify how much noise in the counts, both within concentrations and between concentrations, affects the precision and recall of each method.
